# A nationwide, population-based, long-term follow-up study of repeated self-harm in Taiwan

**DOI:** 10.1186/1471-2458-12-744

**Published:** 2012-09-06

**Authors:** Chi-Hsiang Chung, Ching-Huang Lai, Chi-Ming Chu, Lu Pai, Senyeong Kao, Wu-Chien Chien

**Affiliations:** 1 Graduate Institute of Life Sciences, National Defense Medical Center, No. 161, Section 6, Min-Chuan East Road, Neihu District, Taipei City 11490, Taiwan, Republic of China; 2 School of Public Health, National Defense Medical Center, No. 161, Section 6, Min-Chuan East Road, Neihu District, Taipei City 11490, Taiwan, Republic of China; 3 Taiwan Injury Prevention & Safety Promotion Association, Room 4112, No. 161, Section 6, Min-Chuan East Road, Neihu District, Taipei City 11490, Taiwan, Republic of China

**Keywords:** Repeated self-harm, Follow-up, National Health Insurance Research Database (NHIRD)

## Abstract

**Background:**

Previous follow-up studies of repeated self-harm show that the cumulative risk of repeated self-harm within one year is 5.7%–15%, with females at greatest risk. However, relatively few studies have focused on the Far East. The objective of this study was to calculate the cumulative risk of repeated self-harm over different lengths of follow-up time (3 months, 6 months, and 1–8 years), to determine factors influencing repeated self-harm and to explore the interaction between gender and self-harm methods.

**Methods:**

We used self-harm patient who hospitalized due to first-time self-harm between 2000 and 2007 from 1,230 hospitals in Taiwan. Hospitalization for repeated self-harm among members of this cohort was tracked after 3 months, 6 months, and 1–8 years. Tracking continued until December 31, 2008. We analyzed the cumulative risk and risk factors of repeated self-harm by using negative binomial regression.

**Results:**

Of the 39,875 individual study samples, 3,388 individuals (8.50%) were found to have repeatedly self-harmed. The cumulative risk of repeated self-harm within three months was 7.19% and within one year was 8%. Within 8 years, it was 8.70%. Females were more likely to repeatedly self-harm than males (RR = 1.21, 95% CI = 1.15–1.76). The main method of self-harm was solid or liquid substances (RR = 1.88, 95% CI = 1.23–2.04) or cutting or piercing (RR = 1.36, 95% CI = 1.02–1.82), and in patients with psychiatric disorders were more likely to self-harm (RR = 1.61, 95% CI = 1.48–1.75).

**Conclusions:**

The key time for intervention for repeated self-harm is within three months. Appropriate prevention programs should be developed based on gender differences.

## Background

Repeated self-harm is a major risk factor affecting death from self-harming behaviors [1]. From 3 to 6 months after a self-harm attempt is the high-risk period for repeated self-harm [2]. In Western countries, Owens et al. [3] found that the cumulative risk of self-harm was approximately 15% within one year and approximately 23% within four years [3]. There have been few follow-up studies of repeated self-harm in the Far East area [3,4]. The first follow-up study was conducted by Chen et al.[4], who found that the cumulative risk of repeated self-harm was 5.7% within the first year and 10.5% within five years. They also found that women constituted a high-risk group for repeated self-harm [4].

Because of the paucity of research on the cumulative risk of repeated self-harm in the Far East [3,4] as well as suggestions from previous studies that differences exist in the self-harm methods employed by men and women [5], the researchers felt that an investigation of factors influencing repeated self-harm should consider whether men and women select different methods of self-harm. Furthermore, to resolve the previous research restrictions (small sample size, short tracking time, no controls for physical or mental illness, no investigation of interactions between gender and method of self-harm), this study employed the Taiwan National Health Insurance Research Database (NHIRD) to analyze self-harm hospitalization data from 1,230 hospitals in Taiwan. The goal of this study was to calculate the cumulative risk of repeated self-harm for different follow-up times (3 months, 6 months, and 1–8 years after the first self-harm hospitalization) and to investigate whether there is a correlation between gender and the method of self-harm.

## Methods

### Data source

Taiwan implemented National Health Insurance on March 1, 1995, and the health insurance coverage rate currently exceeds 99%. The National Health Insurance database collects nationwide outpatient/emergency and hospitalization data, and the law requires that all hospitals and clinics report outpatient/emergency and hospitalization expenses to the Bureau of National Health Insurance on a monthly basis. Consequently, National Health Insurance information can serve as representative empirical data in medical- and health-related research fields [6]. Researchers are required to pass a detailed review by a professional peer review committee before they can use the Taiwan National Health Insurance Research Database. Because patients’ identities are encrypted in the database, this study did not infringe on patients’ right to privacy. This study used inpatient expenditures (by admissions) and medical organization data (registry for contracted medical facilities) from the health insurance database. The variables provided in the database included inpatient age, gender, whether the patient was from a low-income family, dates of admission and discharge, care location, hospital level, department, whether surgical procedures were employed, medical expenditures, diagnosis of disease or injury (in accordance with the ICD-9-CM N-Code), and cause of injury (in accordance with the ICD-9-CM E-Code) [6].

**Figure 1:**
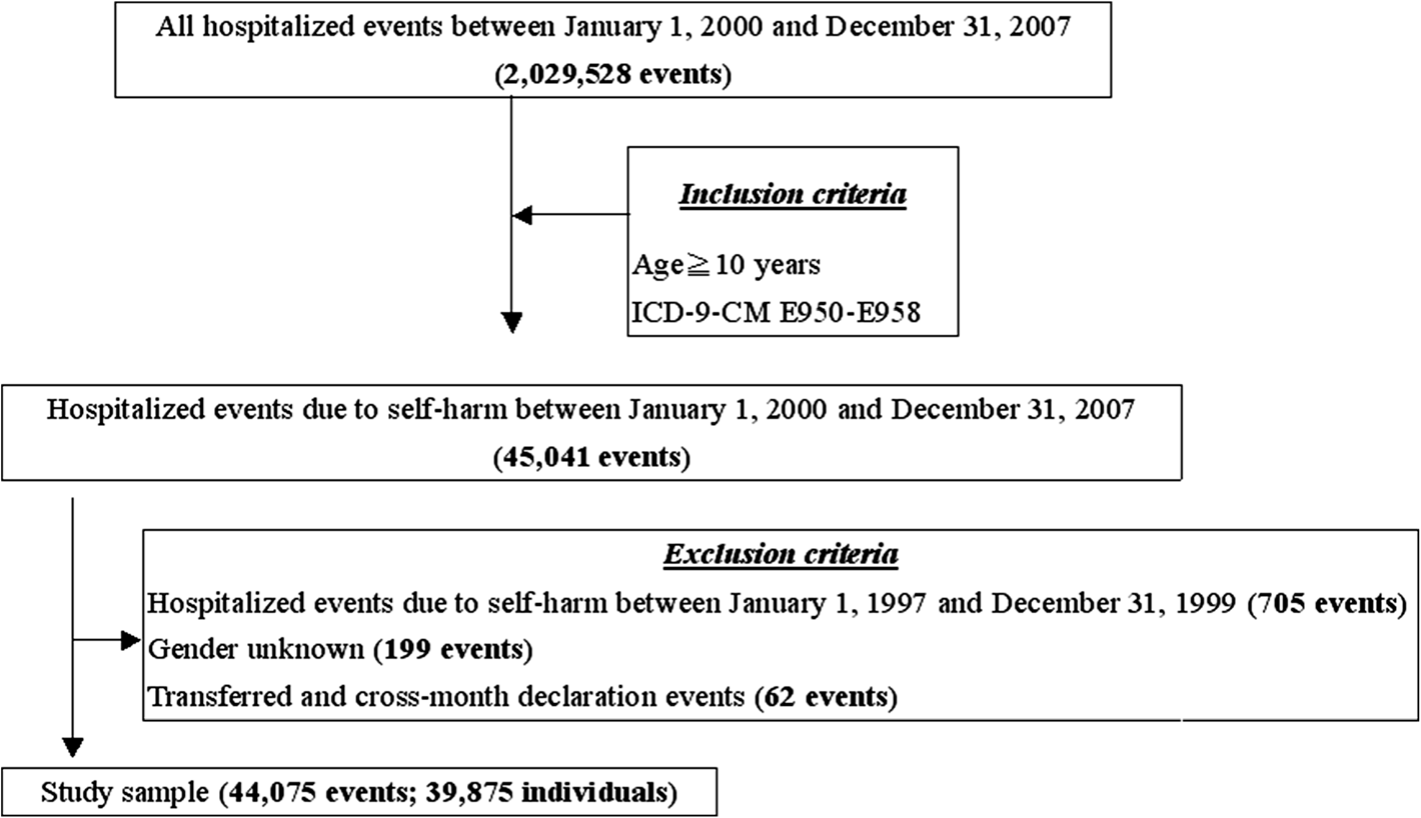
Flowchart of study sample selection from national health insurance research database in Taiwan.

In this prospective cohort study, persons hospitalized due to first-time self-harm in the Taiwan area between 2000 and 2007 were used as the initial cohort, and the sample selection of the in this study are shown in Figure 1. Among 2,029,528 events of all hospitalized data from January 1, 2000 to December 31, 2007, 45,041 events consisted of person aged 10 and over who were hospitalized due to self-harm (E950-E958). After eliminating persons hospitalized due to self-harm from January 1, 1997 to December 31, 1999 (705 events) and persons of unknown gender (199 events) and after deleting cases in which the patient was transferred between hospitals or was hospitalized across different months causing the case to be reported multiple times (62 events), a total of 44,075 events of self-harm inpatient data remained, which was reduced to 39,875 individuals. Hospitalization for repeated self-harm among members of this cohort was tracked after 3 months, 6 months, and 1-8 years. Tracking continued until December 31, 2008.

### Variable definitions

In accordance with the definitions of the World Health Organization (WHO), suicidal actions that do not result in death are called “attempted suicide” (a term common in the United States), “parasuicide” and “deliberate self-harm” (terms common in Europe). “Reoccurrence of self-harm” indicates repeated suicidal actions [7]. In this study, we define “deliberate self-harm” as hospitalization due to self-harm. We categorized “reoccurrence of self-harm” into cases without repetition of self-harm (the patient attempted to self-harm only once during the research period) and repeated self-harm (the patient was hospitalized two or more times for self-harm during the research period).

Demographic data for the first batch of self-harm inpatients included gender (male and female), age (four groups: <25 years, 25–44 years, 45–64 years, and 65 years), method of self-harm (methods were classified into nine groups in accordance with the ICD-9-CM E-Code: E950 solid or liquid substances, E951 gases in domestic use, E952 gases or vapors, E953 hanging, E954 drowning, E955 firearms, E956 cutting or piercing implements, E957 jumping from building, and E958 other means), low-income family (two groups consisting of patients from low-income and non-low-income families), catastrophic illness (two groups consisting of patients with/without catastrophic illness, such as cancers, Injury Severity Score ≧16, and rare diseases), and psychiatric disorders (two groups consisting of patients with/without psychiatric disorders in accordance with the ICD-9-CM N-Code 209-319).

The Abbreviated Injury Scale (AIS) is an anatomically based consensus-derived global severity scoring system that classifies each injury in every body region according to its relative severity on a six point ordinal scale (1: minor, 2: moderate, 3: serious, 4: severe, 5: critical, and 6: maximal, currently untreatable); and the Injury Severity Score (ISS) is an anatomical scoring system that provides an overall score for patients with multiple injuries. Each injury is assigned an Abbreviated Injury Scale (AIS) score and is allocated to one of six body regions (head, face, chest, abdomen, extremities, and external). Only the highest AIS score in each body region is used. The 3 most severely injured body regions have their score squared and added together to produce the ISS score, and the ISS scores ranges from 1 to 75 [8]. In Taiwan, patients with ISS≧16 were defined as catastrophic injury.

### Statistical analysis

This study investigated whether subjects were hospitalized due to self-harm 3 months, 6 months, and 1–8 years after first being hospitalized. Tracking of the subjects continued until December 31, 2008. Based on the research of Chen et al. [4], this study calculated the risk of repeated self-harm based on the individual (only the first repeated self-harm was counted, and individuals could contribute to the numerator in only one annual follow-up period), the risk of repeated self-harm based on episode (any repeated self-harm was counted, and an individual could contribute to the numerator in more than one annual follow-up period), cumulative risk of repeated self-harm, and 95% confidence interval (CI) for the different follow-up times to compare our results with Chen’s [4]. Furthermore, this study drew Kaplan-Meier curves of the cumulative risk of repeated self-harm for all subjects and for male and female subjects and used the log-rank test to check for differences between men and women (setting P < 0.05 as the threshold of significant variance).

In previous research on factors predicting repeated self-harm based on the individuals, some scholars have used Cox’s regression [9] or Poisson regression [10] to identify factors affecting this behavior. However, there were differences in the frequency of self-harm attempts and methods during the research period. The dependent variable belonged to a count outcome variable, so other studies recommend the use of negative binomial regression analysis in a generalized estimating equation (GEE) model [4,11]. This study used SPSS 20.0 software, employed negative binomial regression as an analytical method, and controlled for environmental factors (season and area), hospital-related factors (hospital level, department, the use of surgical procedures, length in days, and medical expenditures), and length of follow-up time. After incorporating terms indicating gender and self-harm method into the model, the study identified factors affecting repeated self-harm (setting P < 0.05 as the threshold of significant variance).

## Results

### Characteristics of study sample

Among the 39,875 persons in this study, 96.40% were tracked for 3 months, 92.97% were tracked for 6 months, 90.95% were tracked for one year, 81.09% were tracked for two years, 69.93% were tracked for three years, 58.17% were tracked for four years, 46.50% were tracked for five years, 34.31% were tracked for six years, 22.70% were tracked for seven years, and 10.98% were tracked for eight years. A total of 3,388 persons in the sample (8.50%) had repeatedly attempted self-harm. Within this group, 2,810 persons repeated self-harm once (hospitalized twice for self-harm during the research period), 435 repeated self-harm twice, 93 repeated self-harm three times, 26 repeated self-harm four times, 14 repeated self-harm five times, 4 repeated self-harm six times, 5 repeated self-harm seven times, and one person repeated self-harm eight times. The average number of times of repeated self-harm was 1.24 ± 0.64.

Females accounted for more cases of repeated self-harm than males. Patients aged 25–44 accounted for approximately one-half of cases. The top three methods used by persons attempting repeated self-harm were solid or liquid substances, cutting or piercing, and gases or vapors. Of the persons who attempted repeated self-harm, 4.43% were from low-income families, 19.86% had catastrophic illness, and more than half had psychiatric disorders (Table 1).

**Table 1:** Characteristics of study sample (N = 39,875)

**Repetition Characteristics**	**No**		**One or more**		**Total**	
	**N**	**(%)**	**N**	**(%)**	**N**	**(%)**
Gender						
Male	16,892	(46.30)	1,492	(44.04)	18,384	(46.10)
Female	19,595	(53.70)	1,896	(55.96)	21,491	(53.90)
Age group (years)						
<25	6,725	(18.43)	532	(15.70)	7,257	(18.20)
25-44	16,222	(44.46)	1,683	(49.68)	17,905	(44.90)
45-64	7,944	(21.77)	729	(21.52)	8,673	(21.75)
≧65	5,596	(15.34)	444	(13.11)	6,040	(15.15)
Methods						
E950 solid or liquid	23,767	(65.14)	2,298	(67.83)	26,065	(65.37)
E951 gases in domestic use	169	(0.46)	10	(0.30)	179	(0.45)
E952 other gases or vapors	2,917	(7.99)	310	(9.15)	3,227	(8.09)
E953 hanging	660	(1.81)	50	(1.48)	710	(1.78)
E954 drowning	226	(0.62)	22	(0.65)	248	(0.62)
E955 firearms	67	(0.18)	8	(0.24)	75	(0.19)
E956 cutting and piercing	6,463	(17.71)	455	(13.43)	6,918	(17.35)
E957 jumping	717	(1.97)	106	(3.13)	823	(2.06)
E958 other means	1,501	(4.11)	129	(3.81)	1,630	(4.09)
Low-income family						
Yes	706	(1.93)	150	(4.43)	856	(2.15)
No	35,781	(98.07)	3,238	(95.57)	39,019	(97.85)
Catastrophic illness						
Yes	2,734	(7.49)	673	(19.86)	3,407	(8.54)
No	33,753	(92.51)	2,715	(80.14)	36,468	(91.46)
Psychiatric disorders						
Yes	14,690	(40.26)	1,881	(55.52)	16,571	(41.56)
No	21,797	(59.74)	1,507	(44.48)	23,304	(58.44)
Total	36,487		3,388		39,875	

### Cumulative risk of repeated self-harm

**Figure 2:**
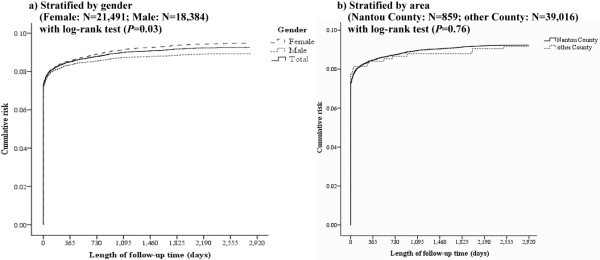
**Kaplan-Meier curves for cumulative risk of repeated self-harm.** (**a**) Stratified by gender. (**b**) Stratified by area.

Figure 2 uses Kaplan-Meier curves to display the cumulative risk of repeated self-harm at different follow-up times for the cases in this study. It can be seen that the inpatients were at greatest risk of repeated self-harm within 3 months. Furthermore, persons hospitalized due to self-harm had a cumulative risk of repeated self-harm of 7.19% within 3 months, 7.77% within 6 months, 8.00% within one year, and 8.70% within eight years (Table 2).

**Table 2:** Risk of repeated self-harm tracked after 3 months, 6 months, and 1-8 years (N = 39,875)

**Time of repeated self-harm**	**Based on individuals**	**Based on episodes**	**Cumulative risk**
Tracked after 3 months			
Events	2,802	5,272	2,802
Risk % (95% CI)	7.19 (6.93–7.45)	7.23 (6.48–7.57)	7.19 (6.93–7.45)
Tracked after 6 months			
Events	225	1,479	3,027
Risk % (95% CI)	0.58 (0.50–0.66)	1.24 (1.13–1.35)	7.77 (7.50–8.08)
Tracked after 1 year			
Events	90	190	3,117
Risk % (95% CI)	0.23 (0.18–0.28)	0.49 (0.42–0.56)	8.00 (7.73–8.60)
Tracked after 2 years			
Events	98	220	3,215
Risk % (95% CI)	0.25 (0.20–0.30)	0.57 (0.50–0.65)	8.25 (7.97–9.20)
Tracked after 3 years			
Events	75	175	3,290
Risk % (95% CI)	0.19 (0.15–0.24)	0.45 (0.39–0.52)	8.44 (8.16–9.68)
Tracked after 4 years			
Events	30	62	3,320
Risk % (95% CI)	0.08 (0.05–0.11)	0.16 (0.12–0.20)	8.52 (8.24–9.85)
Tracked after 5 years			
Events	30	92	3,350
Risk % (95% CI)	0.08 (0.05–0.11)	0.24 (0.19–0.29)	8.60 (8.32–10.10)
Tracked after 6 years			
Events	24	68	3,374
Risk % (95% CI)	0.06 (0.04–0.09)	0.18 (0.14–0.22)	8.66 (8.38–10.28)
Tracked after 7 years			
Events	11	24	3,385
Risk % (95% CI)	0.03 (0.01–0.05)	0.06 (0.04–0.09)	8.69 (8.41–10.35)
Tracked after 8 years			
Events	3	6	3,388
Risk % (95% CI)	0.01 (0.01–0.02)	0.02 (0.01–0.03)	8.70 (8.42–10.37)

CI. confidence interval.

The Kaplan-Meier curves for cumulative risk of repeated self-harm by gender at different follow-up times (Figure 2a) reveal that females had a cumulative risk of repeated self-harm of 8.82% within eight years (95% CI = 8.54%–9.20%), whereas males had a cumulative risk of 8.12% (95% CI = 7.72%–8.51%). Furthermore, log-rank test results (P = 0.03) reveal that females have a higher cumulative risk of repeated self-harm than do males.

### Risk factors of repeated self-harm

Because of the excessive paucity of cases involving certain methods of self-harm, the original nine methods of self-harm were reduced to six groups: solid or liquid substances (E950), gases in domestic use (E951), other gases or vapors (E952), cutting or piercing implements (E956), violent methods (E953 hanging, E954 drowning, E955 firearms, E957 jumping from building), and other means (E958). Within the model as whole, females had a greater risk of repeated self-harm than males. Most persons who attempted repeated self-harm used solid or liquid substances or cutting methods, and patients with psychiatric disorders had a relatively high risk of repeated self-harm. Because the interaction terms of gender and self-harm method were significant, gender will be discussed separately in the following section (Table 3).

**Table 3:** **Risk factors of repeated self-harm by using negative binomial regression model**^**a**^

**Model Characteristics**	**Total (N = 39,875)**		**Female (N = 21,491)**		**Male (N = 18,384)**	
	**Adjusted RR**^**b**^**(95% CI)**	**P**	**Adjusted RR**^**b**^**(95% CI)**	**P**	**Adjusted RR**^**b**^**(95% CI)**	**P**
Gender						
Male	Reference					
Female	1.21 (1.15–1.76)	0.04				
Age group (years)						
<25	Reference		Reference		Reference	
25–44	0.83 (0.72–1.26)	0.16	0.94 (0.92–1.33)	0.06	0.79 (0.63–1.29)	0.29
45–64	0.86 (0.75–1.08)	0.12	0.90 (0.76–1.06)	0.21	0.80 (0.66–1.69)	0.33
≧65	0.63 (0.54–1.73)	0.33	0.96 (0.46–1.70)	0.33	0.56 (0.52–1.45)	0.43
Methods						
Violent methods ^c^	Reference		Reference		Reference	
E950 solid or liquid	1.88 (1.23–2.04)	<0.01	2.02 (1.29–2.18)	<0.01	1.76 (1.58–2.83)	<0.01
E951 gases in domestic use	1.56 (0.81–1.97)	0.24	1.86 (0.76–2.13)	0.39	1.36 (0.89–2.01)	0.20
E952 other gases or vapors	1.80 (0.76–2.09)	0.16	2.24 (0.88–3.04)	0.34	1.66 (0.95–2.29)	0.06
E956 cutting and piercing	1.36 (1.02–1.82)	0.03	1.38 (1.02–1.83)	0.02	1.26 (0.69–1.51)	0.17
E958 other means	0.84 (0.66–1.40)	0.44	0.85 (0.54–1.54)	0.54	0.81 (0.64–1.34)	0.43
Low-income family						
Yes	1.86 (0.91–2.28)	0.25	2.12 (0.62–2.77)	0.68	1.55 (0.82–2.13)	0.23
No	Reference		Reference		Reference	
Catastrophic illness						
Yes	1.10 (0.97–1.25)	0.14	1.21 (0.82–1.44)	0.07	0.97 (0.80–1.18)	0.76
No	Reference		Reference		Reference	
Psychiatric disorders						
Yes	1.61 (1.48–1.75)	<0.01	1.70 (1.52–1.90)	<0.01	1.49 (1.31–1.69)	<0.01
No	Reference		Reference		Reference	

RR. relative risk, CI. confidence interval.

^a^All of the variables were significant (P < 0.05) in univariate analysis. The interaction between gender and methods was significant (P = 0.01).

^b^Adjusted for environmental factors (season and area), hospital-related factors (levels of hospital, department, whether surgical procedures were employed, length of days, and medical expenditures), and length of follow-up time.

^c^Including E953 (hanging), E954 (drowning), E955 (firearms), and E957 (jumping).

Among females, the majority of persons attempting repeated self-harm used methods involving solid or liquid substances (RR = 2.02, 95% CI = 1.29–2.18) or cutting (RR = 1.38, 95% CI = 1.02–1.83). Patients with psychiatric disorders had a relatively high risk of repeated self-harm (RR = 1.70, 95% CI = 1.52–1.90). In the male model, the majority of persons who attempted repeated self-harm used solid or liquid substances (RR = 1.76, 95% CI = 1.58–2.83) or other gases or vapors (RR = 1.66, 95% CI = 0.95–2.29). Patients with psychiatric disorders also had a relatively high risk of repeated self-harm (RR = 1.49, 95% CI = 1.31–1.69) (Table 3). This study also performed pivot table analysis of gender and methods of repeated self-harm and found that the top three methods used by females in their first repeated self-harm attempt were solid or liquid substances, cutting or piercing implements, and gases or vapors, whereas the top three methods used by males were solid or liquid substances, gases or vapors, and cutting or piercing implements (Table 4).

**Table 4:** Methods of repeated self-harm by gender (N = 3,388)

**Methods of repeated self-harm**	**Female**		**Male**		**Total**	
	**N**	**(%)**	**N**	**(%)**	**N**	**(%)**
E950 solid or liquid	1,334	(70.36)	964	(64.61)	2,298	(67.83)
E951 gases in domestic use	6	(0.32)	4	(0.27)	10	(0.30)
E952 other gases or vapors	164	(8.65)	146	(9.79)	310	(9.15)
E953 hanging	30	(1.58)	20	(1.34)	50	(1.48)
E954 drowning	12	(0.63)	10	(0.67)	22	(0.65)
E955 firearms	5	(0.26)	3	(0.20)	8	(0.24)
E956 cutting and piercing	340	(17.93)	115	(7.71)	455	(13.43)
E957 jumping	60	(3.16)	46	(3.08)	106	(3.13)
E958 other means	45	(2.37)	84	(5.63)	129	(3.81)
Total	1,896		1,492		3,388	

## Discussion

### Cumulative risk of repeated self-harm

This study found that persons hospitalized due to self-harm were at greatest risk of repeated self-harm during the first 3 months, followed by the first 6 months. This finding is consistent with the findings of prior studies suggesting that the risk of repeated self-harm is highest within 3-6 months after a self-harm attempt [2]. The trans-theoretical model proposed by DiClemente and Prochaska et al. in 1982 suggested that even when intervention methods and strategies are used to change a person’s behavior, the process of change requires at least 6 months to be successful [12]. This may be why the period of highest risk of repeated self-harm is 3-6 months after a self-harm attempt.

This study found that persons hospitalized due to self-harm had an 8.60% cumulative risk of repeated self-harm during a five-year period. This number is lower than the value obtained by Chen et al. (10.5%) in their study of cases in Taiwan from July 2000 to February 2003, which were tracked until the end of December 2005 [4]. This discrepancy may be because, although the scope of the current study encompassed all of Taiwan, the study by Chen et al. covered only Nantou County [4]. In a stratified analysis of our study showed that the cumulative risk of repeated self-harm in Nantou County (8.65%, 95% CI = 8.42–10.07) was slightly higher than that of rest in Taiwan (8.57%, 95% CI = 8.32–10.04) tracked after 5 years (Figure 2b). Nantou County suffered severe damage in the Chi-Chi earthquake of 1999 and experienced an 81.09% increase in self-harm from 1998 to 2001, which was far higher than the equivalent increase in other areas of Taiwan (24.97%) [13]. This may be why the level of risk found by Chen et al. was higher than that found in this study.

The 8.70% cumulative risk of repeated self-harm among persons hospitalized due to self-harm within five years in this study was lower than the result found by Owens (15% risk within one year and approximately 23% risk within four years) in research on Western countries [3]. This phenomenon might be attributable to regional differences because the self-harm death rate is uniformly higher in Asian countries than in the West [14]. Thus, there are fewer self-harm attempts in Asia than in the West, and the risk of repeated self-harm is lower in Asia than in Western countries [4].

### Risk factors of repeated self-harm

This study found that females had a higher risk of repeated self-harm than did males (RR = 1.21). This study’s findings were similar to those obtained in Australia [15] and Brazil [16], where females were also found to have a higher risk of repeated self-harm than males (the Australian study found that females had 1.9 times the repeated self-harm risk of males [15], whereas the Brazilian study found that females had 2.7 times the risk of males [16]). However, studies in Britain [17] and Sri Lanka [18] found no significant variance in the repeated self-harm risk of the two genders. Furthermore, according to a report issued by the WHO, with the exception of rural areas of China, males had a higher self-harm mortality rate than females in other parts of the world [7], which implies that females have a higher rate of self-harm attempts than males. In addition, females were more likely to seek assistance from medical organizations [19], which may explain this study’s finding of a relatively high risk of repeated self-harm among females.

This study found that a majority of persons attempting repeated self-harm choose solid or liquid substances (RR = 1.88) or cutting or piercing implements (RR = 1.36). This finding suggests that these persons usually do not select methods that are most likely to be fatal. This phenomenon is consistent with the results of a cohort study in Britain, which found that cutting implements and drugs were the most commonly used methods in repeated self-harm attempts [20]. An Australian study found that persons attempting self-harm by taking poison had a relatively high risk of repeated self-harm [11]. Moreover, this study identified an interaction (P = 0.01) between gender and methods of repeated self-harm. When the genders were analyzed separately, it was discovered that females’ preferred methods of repeated self-harm consisted largely of solid or liquid substances (RR = 2.02) or cutting or piercing implement methods (RR = 1.38), whereas males predominately chose solid or liquid substances (RR = 1.76) or other gases or vapors (RR = 1.66). It appears that males choose more violent methods of self-harm than do females. Although both males and females preferentially selected cutting methods, males may tend to cut themselves more aggressively, causing deeper wounds and greater injury. One study showed that during the period of 1986-2007 in Taiwan, males who used cutting implements to attempt self-harm had a higher mortality rate than females (0.32 per 100,000 vs. 0.10 per 100,000) [5].

This study further found that regardless of gender, patients with psychiatric disorders had a relatively high risk of repeated self-harm (overall model RR = 1.61; male model RR = 1.49; female model RR = 1.70). According to a British study, persons with mental illness had a relatively high likelihood of repeated self-harm [21]. A study in the US similarly indicated that the greater the number of a person’s concurrent mental illnesses, the higher the incidence of repeated self-harm [22], which was consistent with the results of this study. Because the symptoms of mental illness (such as hallucinations) might cause patients extreme distress, such patients were more likely to resort to suicide. Furthermore, after a patient with mental illness attempts self-harm, mood swings may cause self-harm again [23]. As a result, patients with psychiatric disorders had a relatively high risk of repeated self-harm.

### Strengths and limitations

This study makes some contributions to the literature. First, because there was very few follow-up studies of repeated self-harm in the Far East area, this study may serve as a reference for efforts to design relevant studies in other Far Eastern countries, and the research findings could be used in international comparisons. Second, because this study used nationwide data from Taiwan’s National Health Insurance database, there should be no sampling bias problems. Furthermore, the fact that this study examined 39,875 cases and tracked cases for up to eight years allowed it to overcome the shortcomings of insufficient sample size and insufficiently long tracking time found in many previous studies. Third, in addition to providing the gender, age, and self-harm methods of persons attempting repeated self-harm, the National Health Insurance database also records patients’ physical and mental illnesses and other medical care-related information. Consequently, these data allowed potential confounding factors to be controlled when deriving self-harm factors. Fourth, previous studies found that self-harm methods differ between urban and rural populations [24,25]. The National Health Insurance database provided areas of self-harm that allowed the level of urban city to be adjusted when deriving self-harm factors. Finally, this study verifies the interaction between the gender of self-harm patients and their self-harm methods, which would facilitate the drafting of appropriate controlled projects focusing on the different genders.

This study also had some limitations. First, the study was limited to the variables that could be obtained from the health insurance database. It could not investigate certain factors that might influence repeated self-harm, such as educational level, marital status, religious beliefs, occupation, and family history of self-harm [7,14]. Second, although this study chose to track first-time self-harm inpatient cases occurring after 2000, self-harm cases occurring from January 1, 1997 to December 31, 1999 had to be excluded due to a lack of health insurance data. As a result, whether inpatient cases prior to 1997 involved self-harm could not be confirmed. Third, the National Health Insurance database did not provide clinical biochemistry data and the Glasgow Coma Scale (GCS). Therefore, we used medical-related factors (whether patients received surgical procedures, length of days, and medical expenditures) as indicators of the severity of self-harm and controlled for them when deriving self-harm factors. Fourth, this study used inpatient data exclusively and could not obtain information on cases in which injury was mild enough to not require care or the patient received only outpatient/emergency care. Although the National Health Insurance database contained data on outpatient/emergency care, these data provided only the diagnosis of disease or injury (in accordance with the ICD-9-CM N-Code) but did not record the cause of injury (in accordance with the ICD-9-CM E-Code) [6]. Consequently, outpatient/emergency care data could not be used to analyze self-harm methods, and our study only could represent the population with greater severity of consequence after self-harm. Fifth, the mortality data could not be linked with the National Health Insurance database in Taiwan because of patient privacy issues. Therefore, we were unable to explore censored data due to unnatural death/suicide or other competing risks. Finally, because previous research has suggested that many self-harm cases in Taiwan could be attributed to other causes (unintentional injury, uncertain intentionality) [26], the results of this study might reflect underestimates.

## Conclusions

Persons hospitalized due to self-harm are at greatest risk (7.19%) of repeated self-harm within three months. Therefore, this is the key time for intervention. We identified interactions between gender and self-harm method: females generally use solid or liquid substances and cutting or piercing, whereas males use solid or liquid substances and other gases or vapors. Thus, appropriate prevention programs should be developed that consider gender differences. Further studies are needed to discuss the number of repeated self-harm.

## Competing interests

The authors declare that they have no competing interests.

## Authors’ contributions

WCC contributed to the study design, obtained the data and commented on the interpretation. CHC contributed to the interpretation of the data and drafted the paper. CHL contributed to the interpretation of the data. CMC, LP, and SK provided suggestions for revision of the manuscript. All authors read and approved the final manuscript.

## Pre-publication history

The pre-publication history for this paper can be accessed here:

http://www.biomedcentral.com/1471-2458/12/744/prepub
